# Health inequity focus in pandemic preparedness and response plans

**DOI:** 10.2471/BLT.21.287580

**Published:** 2022-02-01

**Authors:** Oscar J Mujica, Christine E Brown, Cesar G Victora, Peter O Goldblatt, Jarbas Barbosa da Silva

**Affiliations:** a Pan American Health Organization, 525 23rd St. N.W. Washington, DC 20037, United States of America.; b World Health Organization European Office for Investment for Health and Development, Venice, Italy.; c International Center for Health Equity, Federal University of Pelotas, Pelotas, Brazil.; d Institute of Health Equity, University College London, London, England.

The *International Health Regulations, 2005 revision*; IHR (2005), are a binding instrument of international law. The IHR (2005) are therefore key for pandemic preparedness and response, leading global efforts for the prevention and control of the spread of disease. Since the IHR (2005) entered into force on 15 June 2007, six public health emergencies of international concern have been declared. 

The coronavirus disease 2019 (COVID-19) emergency has shown fractures in societies, exposing deeply rooted social inequities, discrimination and health gradients across human populations, between and within countries. The Independent Panel on Pandemic Preparedness and Response, established by the World Health Organization in September 2020, concluded that COVID-19 is a pandemic of inequalities and inequities.^1^

The COVID-19 pandemic has compounded pre-existing social inequities in health and created new vulnerabilities in three ways. First, the health consequences of COVID-19 have unequal socioeconomic effects across population groups. Second, containment measures have not affected all groups in the same way. Third, the pandemic has bidirectional effects ‒ between the unequal socioeconomic consequences of COVID-19 and health inequities that are not related to COVID-19.^2^ The pandemic has revealed that preparedness and response plans had not adequately anticipated or considered the likely worsening of these inequalities.

The exposure of social and health inequities calls for the inclusion of health inequity considerations into pandemic preparedness and response plans, both for the potential next COVID-19 waves and for future pandemics and other emergencies. Pandemic preparedness and response plans differ from those associated to long-term recovery and build-back plans, such as those related to the sustainable development goals. Actions implemented as part of response plans can inform and contribute to longer term development goals. However, the goal of leaving no one behind will not be reached if the international community remains indifferent to inequities. Disaster recovery responses must contemplate transformative change and avoid engendering feedback loops that create greater vulnerability^3^ and amplify and perpetuate long-standing socioeconomic, gender, racial-ethnic and health inequities.

Equity is currently the focus of attention for policy-makers, particularly within the context of pandemic preparedness and response plans. The establishment of the Presidential COVID-19 Health Equity Task Force by President Biden, with 55 prioritized recommendations, implementation plan and accountability framework,^4^ is a case in point. Another encouraging example is the framework proposed by two Canadian national collaborating centres for public health, *Measuring what counts in the midst of the COVID-19 pandemic: equity indicators for public health*,^5^ which covers 11 essential dimensions of a pandemic preparedness and response plan with 67 health equity indicator prompts.

The international community increasingly recognizes the need for better governance of the interdependencies between health, social, environmental and economic systems. Such governance should explicitly place equity and well-being for all at the heart of the COVID-19 sustainable response. The mechanism to achieve such good governance in practice is embodied in the recommendations of the Pan-European Commission on Health and Sustainable Development, *Drawing light from the pandemic*,^6^ and in the Asian Development Bank report *Wellness in worrying times*.^7^ The approach of the 2019 *Healthy, prosperous lives for all: the European Health Equity Status Report*^8^ has inspired countries and territories such as Italy, North Macedonia, Slovenia and Wales to explicitly put health equity at the heart of the COVID-19 sustainable response. The *Welsh health equity status report* initiative^9^ frames the pandemic response around investment in five essential conditions and gender-responsive policy action areas for health equity: health services; income security and social protection; living conditions; human and social capital; and employment and working conditions. The initiative is closely aligned with the United Nations socioeconomic immediate response pillars, emphasizing that governance for health equity is mainstreamed into policy coherence, accountability, social participation and community empowerment approaches.

However, perhaps the most politically significant event has been the call, on 30 March 2021, of 26 heads of state for a new international pandemic treaty, intended to point at the high-level political action needed to protect the world from future health crises.^10^ The momentum generated by this joint call prompted the creation of a Member States Working Group within the World Health Assembly, tasked with assessing the benefits of such a treaty. In the working group’s report, equity is the first and most critically important aspect of preparedness and response not within the scope of the IHR (2005) that may be best addressed by the treaty.^11^ On 1 December 2021, the World Health Assembly passed the decision to establish an intergovernmental negotiating body to strengthen pandemic prevention, preparedness and response,^12^ effectively paving the way for the inclusion of the much-needed health inequity focus in such plans.
